# Honey bee‐collected pollen in agro‐ecosystems reveals diet diversity, diet quality, and pesticide exposure

**DOI:** 10.1002/ece3.3178

**Published:** 2017-08-05

**Authors:** Megan J. Colwell, Geoffrey R. Williams, Rodger C. Evans, Dave Shutler

**Affiliations:** ^1^ Department of Biology Acadia University Wolfville NS Canada; ^2^ Department of Biology Dalhousie University Halifax NS Canada; ^3^ Institute of Bee Health Vetsuisse Faculty University of Bern Bern Switzerland; ^4^ Agroscope, Swiss Bee Research Centre Bern Switzerland; ^5^ Present address: Department of Entomology University of Manitoba Winnipeg MB Canada R3T 2N2; ^6^ Present address: Department of Entomology & Plant Pathology Auburn University Auburn AL 36849 USA

**Keywords:** *Apis mellifera*, floral diversity, honey bees, neonicotinoids, nutrition, pesticides, pollen

## Abstract

European honey bees *Apis mellifera* are important commercial pollinators that have suffered greater than normal overwintering losses since 2007 in North America and Europe. Contributing factors likely include a combination of parasites, pesticides, and poor nutrition. We examined diet diversity, diet nutritional quality, and pesticides in honey bee‐collected pollen from commercial colonies in the Canadian Maritime Provinces in spring and summer 2011. We sampled pollen collected by honey bees at colonies in four site types: apple orchards, blueberry fields, cranberry bogs, and fallow fields. Proportion of honey bee‐collected pollen from crop versus noncrop flowers was high in apple, very low in blueberry, and low in cranberry sites. Pollen nutritional value tended to be relatively good from apple and cranberry sites and poor from blueberry and fallow sites. Floral surveys ranked, from highest to lowest in diversity, fallow, cranberry, apple, and blueberry sites. Pesticide diversity in honey bee‐collected pollen was high from apple and blueberry sites and low from cranberry and fallow sites. Four different neonicotinoid pesticides were detected, but neither these nor any other pesticides were at or above LD_50_ levels. Pollen hazard quotients were highest in apple and blueberry sites and lowest in fallow sites. Pollen hazard quotients were also negatively correlated with the number of flower taxa detected in surveys. Results reveal differences among site types in diet diversity, diet quality, and pesticide exposure that are informative for improving honey bee and land agro‐ecosystem management.

## 1 INTRODUCTION

Insect pollination contributed an estimated €153 billion to agriculture worldwide in 2005 (Gallai, Salles, Settele, & Vaissièrre, 2009). Although there are many insect pollinators, European honey bees (*Apis mellifera*; hereafter, honey bees) are arguably the single most recognizable and most economically important commercial species (Moritz et al., 2010). Honey bees are generalist pollinators of a wide variety of plant taxa (Winston, 1991). Accordingly, one‐third of human diets are estimated to be dependent on honey bees (Delaplane & Mayer, 2000). Pollination services are how industrial apiculture generates the majority of its profits; industrialization of apiculture means that honey bees spend extended intervals in a single crop, and are transported successively from monoculture to monoculture, each of which contains a new suite of flowers and often a new suite of agricultural pesticides. Although honey bee populations have been increasing for the past five decades globally, above average annual winter colony mortality has occurred regularly since 2007 in both North America and Europe, with serious attendant economic consequences (Currie, Pernal, & Guzmán‐Novoa, 2010; De la Rúa, Jaffé, Dall'Olio, Muñoz, & Serrano, 2009; vanEngelsdorp & Meixner, 2010). Effects of parasites [following Anderson and May (1982), this term includes viral, bacterial, and fungal microparasites] on honey bee losses have been well studied (Higes et al., 2008; Berthoud et al. 2010; Guzman‐Novoa et al. 2010; Aronstein et al. 2012; Dainat & Neumann 2013). However, the extent to which successive restricted diets within monocultures and successive exposure to agricultural pesticides are contributing to poor health is less well understood; these latter variables were foci in this study.

Proper nutrition enhances as well as maintains good health and colony sustainability (Brodschneider & Crailsheim, 2010). Honey bee foragers collect nectar, pollen, and water from flowering plants (Winston, 1991). Importantly, pollen is honey bees' only significant source of protein, lipids, minerals, and vitamins, all of which are necessary for brood‐rearing, normal development, and worker longevity (Cook, Awmack, Murray, & Williams, 2003; Di Pasquale et al., 2016; Mattilla & Otis, 2006; Sagili & Pankiw, 2007; Singh & Singh, 1996; Standifer, 1967). Pollen‐stressed larvae became earlier and poorer foragers, dancers, and had lower mass and longevity as adults compared to normally fed larvae (Schofield & Mattila, 2015). Pollen quality is often quantified by percent protein (e.g., the mean percent of pollen protein in one study was 20.6%; Szczęsna, 2006), but a more biologically relevant measurement can be amino acid composition (Haydak, 1970). Nutritional content of pollen is highly variable among plant species and among geographic regions (Brodschneider & Crailsheim, 2010). Thus, to obtain sufficient nutrition from pollen, honey bees may have to forage on a variety of plant taxa to obtain all their essential nutrients (Singh & Singh, 1996). However, honey bees that are pollinating monocultures may have limited access to alternative sources of pollen, which could lead to nutritional stresses, and this could partially account for overwintering losses (Alaux, Ducloz, Crauser, & Le Conte, 2010; Boucher et al., 2012, 2013; Pernal, 2009, 2010; Pernal et al., 2011). Interestingly, Di Pasquale et al. (2016) found that honey bee health was more affected by pollen quality than pollen diversity. However, when challenged with the microsporidian parasite *Nosema ceranae*, bees fed polyfloral diets lived longer than those on monofloral diets (Di Pasquale et al., 2013). These results suggest that when bees are stressed, pollen diversity may be more important than pollen quality.

Additional concerns for industrial apiculture are pesticides in agriculture; honey bees are sometimes more susceptible to insecticides than are target pests (Claudianos et al., 2006; Scott‐Dupree et al., 1995). Higher susceptibility to pesticide residues (i.e., pesticides and their breakdown products) may be attributed to honey bees having significantly fewer genes in three gene superfamilies that encode detoxifying enzymes (Claudianos et al., 2006). This puts honey bees in danger of lethal and sublethal exposures in agro‐ecosystems (Brittain & Potts, 2011; Bromenshenk, Carlson, Simpson, & Thomas, 1985; Porrini et al., 2003; Krupke et al., 2012). Aside from out‐of‐hive pesticides, beekeepers increasingly have to resort to in‐hive pesticides to control honey bee parasites. Some in‐hive chemicals, such as coumaphos that is used to control *Varroa destructor* mites, can build up in comb wax, prolonging honey bee exposure following application (Martel et al., 2007; Moritz et al., 2010). Risks associated with pesticides in pollen can be quantified with a pollen hazard quotient (PHQ). A PHQ takes into account both the amount and LD_50_ values of each pesticide detected in a sample (Stoner & Eitzer, 2013). PHQs do not take chemical interactions into account and on their own do not resolve the number or lethality of pesticides that are present (e.g., a low PHQ sample may result from a small amount of one low LD_50_ pesticide, or a larger array of tiny amounts of high LD_50_ pesticides). Nevertheless, PHQs are a valuable general metric of pesticide risk to honey bees.

An additional concern is that little is known about potential synergisms among pesticides (but see Johnson, Pollock, & Berenbaum, 2009; Johnson, Dahlgren, Siegfried, & Ellis, 2013), whether in‐hive or out‐of‐hive. To illustrate the pesticide problem, one study of honey bee‐collected pollen (hereafter, pollen) in 23 states and one Canadian province found 98 different pesticide residues, with a mean of 7.1 residues per pollen sample (Mullin et al., 2010). A similar survey in France found the neonicotinoid imidacloprid in 49.4% of samples and had from 1 to 5 different residues in positive samples (testing for up to 41 different residues; Chauzat et al., 2006; also see Porrini et al., 2016). Although direct mortality of honey bees from pesticides is not commonly observed in the developed world, there may be sublethal effects that can affect foraging behavior, colony development, odor discrimination, learning, locomotion, and queen health (Pettis, Collins, Wilbanks, & Feldlaufer, 2004; De la Rúa et al., 2009; Frost, Shutler, & Hillier, 2013; Williamson et al., 2013; Retschnig et al., 2015; Williams et al., 2015; Eiri & Nieh 2012; see Sanchez‐Bayo & Goka, 2014 for a risk assessment of pesticide contaminated‐pollen, nectar, and honey). Sublethal effects are likely to be long term and put colonies at greater risk from stressors such as parasites that otherwise healthy colonies could manage (De la Rúa et al., 2009; Thompson, 2003). Finally, an indirect consequence of herbicides that are sprayed in some crops (e.g., apples, *Malus pumila*) is a possible reduction in noncrop flower taxa, limiting forage diversity for pollinators (Kevan, 1975; Potts et al., 2010).

Consequences of poor nutrition may interact with pesticide exposure. In particular, nutritionally stressed honey bees that have little protein in their bodies are less capable of enzymatically decomposing pesticides (Wahl & Ulm, 1983). The combination of nutritional stress and pesticide exposure may be additive or even synergistic and may be further compounded by stresses from parasites (Doublet, Labarussias, de Miranda, Moritz, & Paxton, 2015; vanEngelsdorp et al., 2009). Therefore, our objectives were to quantify nutritional quality and pesticide residues of pollen to gain insights into real‐world colony exposure. To our knowledge, this is the first study to simultaneously assess diversity, quality, and pesticide contamination of pollen.

## 2 MATERIALS AND METHODS

### 2.1 Data collection

**Figure 1:**
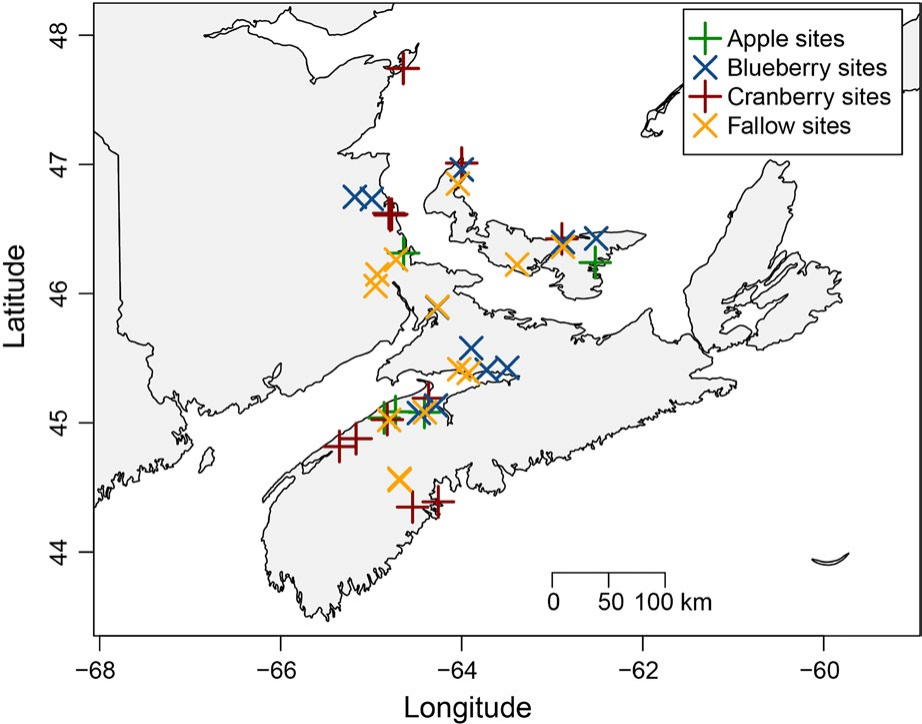
Locations of apiaries sampled for honey bee‐collected pollen in the Maritime Provinces of Canada. Four types of sites were visited: apple (*n *=* *5; green “+”), blueberry (*n *=* *11; blue “x”), cranberry (*n *=* *12; dark red “+”), and fallow sites (*n *=* *13; yellow “x”)

We conducted field work between 26 May and 26 August 2011. We had four site types: apple orchards, blueberry (*Vaccinium angustifolium* and *V. corymbosum*) fields, cranberry (*V. macrocarpon*) bogs, and fallow fields (Table 1). We sampled each of the above site types in the three Maritime provinces of Canada: Nova Scotia (NS), New Brunswick (NB), and Prince Edward Island (PEI; Figure 1). The three crop types were visited in order of bloom. Apples bloom earliest, followed by blueberries with some overlap, and then cranberries. Fallow sites were sampled after the commercial pollination season of these three crop types. Fallow sites were defined as sites without a current honey bee‐pollinated crop in production that year. Fallow sites were usually characterized by pasture lands with grasses and wildflowers.

**Table 1:** Summary of site sampling by province and date ranges for each of the four site types: apple, blueberry, cranberry, and fallow (NS, Nova Scotia; NB, New Brunswick; PEI, Prince Edward Island)

Site type	Sites per province			Sampling period by province		
	NS	NB	PEI	NS	NB	PEI
Apple	3	1	1	26 May–1 June 2011	8–9 June 2011	5–6 June 2011
Blueberry	5	3	3	29 May–18 June 2011	8–22 June 2011	19–24 June 2011
Cranberry	6	4	2	16–17 July 2011	24–25 July 2011	19–20 July 2011
Fallow	6	4	3	17–26 Aug 2011	19–20 Aug 2011	21–22 Aug 2011

Pollen traps were installed on at least three colonies per site. Pollen traps are designed to dislodge pollen loads from honey bee legs as they enter their colony, which fall into collection trays (Delaplane et al., 2013). Traps were installed for at least 24 hr and in some cases for an additional 24 hr to increase the amount or quality (rain homogenized 11.8% of pollen pellet samples) of pollen available for our assays. However, some sites did not have enough pollen for analyses even after additional sampling (Table 2). Minimum volumes of pollen were ~5 ml for pesticide analysis, ~7 ml for plant taxa identification, and ~5 ml for nutritional analyses.

**Table 2:** Summary of pollen collected via traps on colonies, including number of sites and colonies within sites, with a sufficient amount of trapped pollen for any analyses. Also shown is a breakdown of the number of colonies used in pollen taxa identification and number of samples per colony for both nutritional and pesticide residue analyses

Site type	# Sites with pollen	# Colonies with pollen	Taxon ID	Nutrition	Pesticides
			# Colonies used	Samples/colonies	Samples/colonies
Apple	5/5	11/27	9	11/9	21/7
Blueberry	6/11	12/35	10	13/10	22/9
Cranberry	10/12	17/36	14	16/14	21/8
Fallow	10/13	18/40	16	17/16	22/8

After a maximum of 48 hr, traps were removed. Pollen load distribution in trays appeared homogenous as dislodged from bees; however, traps were gently shaken by hand to ensure a relatively homogenous mixture before sampling. Pollen was collected using disposable spoons; fresh spoons were used for each new colony. Pollen samples were first allocated for pesticide analysis and stored in 1.5‐mL microcentrifuge tubes. Additional pollen was then allocated for identification purposes and nutritional analyses and stored in 50‐mL plastic vials. Pesticide samples were stored in a liquid nitrogen dry shipper, whereas other pollen was stored in a cooler on ice packs. Of 175 pollen‐trapping sessions, 123 yielded enough for at least one pesticide sample, and 54 yielded enough for at least one nutrition sample.

Concurrent with pollen trap deployment, floral surveys were performed at each site to identify taxa and estimate available diversity. Four 150‐m transects were oriented in each of the cardinal directions from a central colony. Flowers within 2 m of transect lines were identified using Newcomb (1977).

Voucher flower samples from floral surveys were used to make reference pollen microscope slides (Evans, 1994). Unknown pollen was subsampled for identification using four subsamples of 1g each. Pollen pellets were sorted with a metal probe into lots based on color using a color chart (Hodges, 1974; Dimou & Thrasyvoulou, 2007). An average of 144.9 pollen pellets was sorted in each of the 1‐g samples. Few individual pollen pellets contained more than one floral source; however, these extra sources were treated as contamination because there was always a clear majority or only one source (Dimou, Thrasyvoulou, & Tsirakoglou, 2006). Immediately after sorting, unknown pollen was used to make microscope slides (Evans, 1994). Slides were examined at 400× and photographed using a light microscope and a Canon 5D Mark II camera with a microscope adapter. Photographs of unknown pollen were compared to photographs of known pollen for identification. If an unknown pollen sample was not in the reference slide collection, it was identified using Bassett, Crompton, and Parmelee (1978) and Crompton and Wojtas (1993). Proportion of pollen from crop versus noncrop flowers was calculated.

Fifty‐seven samples of heterogeneous pollen sources (i.e., a representative mix of all pollen taxa from one colony) were sent to AAA Service Laboratories in Oregon, USA, for analysis of pollen nutrition. Samples were analyzed for percent protein content and 20 amino acids with a Hitachi L‐8900 Amino Acid Analyzer with postcolumn, ninhydrin derivatization using high‐pressure liquid chromatography separation (Milio & Loffredo, 1995). Nine of the 20 amino acids are in the ten essential amino acids for honey bee growth, as defined by de Groot (1953). The tenth, tryptophan, was not included due to constraints at the external laboratory.

Eighty‐six heterogeneous pollen samples were sent to the United States Department of Agriculture (USDA) National Science Laboratory in North Carolina, USA, to quantify 174 different compounds (hereafter, residues). Samples were analyzed using a modified popular method for testing food products for residues, called QuEChERS, the quick, easy, cheap, effective, rugged, and safe method, which involves concurrent analysis by gas chromatography/mass spectrometry and liquid chromatography/tandem mass spectrometry (Lehotay, Maštovaká, & Lightfield, 2005; Mullin et al., 2010).

### 2.2 Statistical analyses

Statistical analyses were performed using R (version 3.2.2; R Core Team 2015) with packages *lme4* (Bates, Maechler, Bolker, & Walker, 2015), *multcomp* (Hothorn, Bretz, & Westfall, 2008), *agricolae* (de Mendiburu, 2015), and *piecewiseSEM* (Lefcheck, 2015), and results were plotted with *ggplot2* (Wickham, 2009). Linear mixed‐effects analyses were used to test for differences among site types (site as fixed effect and colony as random effect) in proportion of pollen collected from site crop, percent protein, percent amino acids, number of residues detected, and PHQs. In cases where colony had no effect (e.g., number of taxa in floral surveys by site), ANOVA was used to test for difference among site types. Linear mixed‐effects models were also used for regression analyses (colony as random effect) of number of taxa in floral surveys and number of taxa in collected pollen, number of taxa in floral surveys and PHQ, number of taxa in collected pollen and PHQ, and number of residues detected and PHQ.

Model significance was tested by ANOVA comparisons of fitted models to null models (not including explanatory fixed effect terms, α = 0.05). Post hoc Tukey's tests were performed for significant models comparing differences among sites (Abdi & Williams, 2010). *R* ^2^ values were extracted from regression models.

Sites were treated as independent units, containing three or more sampled colonies. Sample sizes of replicate samples were insufficient to examine repeatability in nutrition and pesticide results. Means are reported ±*SE*.

Pollen hazard quotients of pesticide exposure for each colony were calculated following Stoner and Eitzer (2013):

$$\text{PHQ}=\left(\frac{\left(\text{Detection}\;(\text{ppb})\right)i}{\left({\text{LD}}_{50}\;\left(\frac{\mu \;g}{\mathrm{bee}}\right)\right)i}+\;\frac{\left(\text{Detection}\;(\text{ppb})\right)j}{\left({\text{LD}}_{50}\;\left(\frac{\mu \;g}{\mathrm{bee}}\right)\right)j}+\ldots \;\frac{\left(\text{Detection}\;(\text{ppb})\right)n}{\left({\text{LD}}_{50}\;\left(\frac{\mu \;g}{\mathrm{bee}}\right)\right)n}\right)$$

## 3 RESULTS

### 3.1 Floral diversity

**Figure 2:**
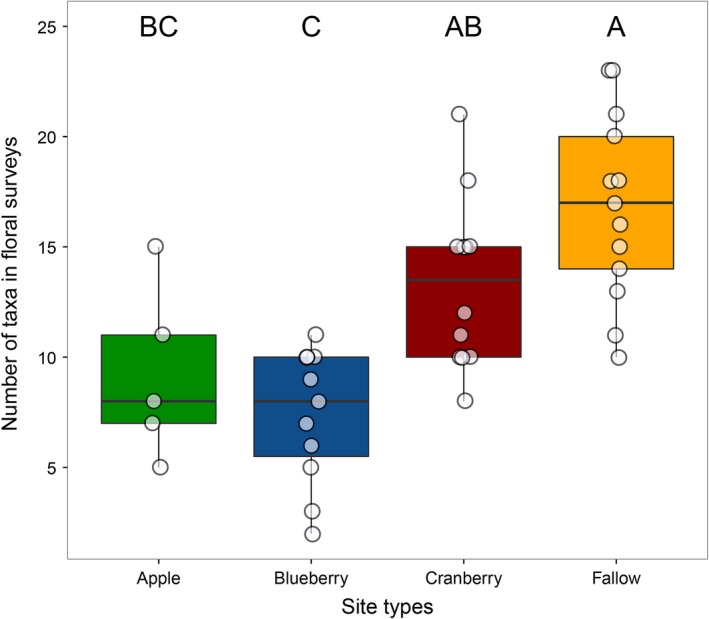
Number of taxa detected in floral surveys differed significantly among site types. Sites with different letters had statistically significant differences in proportion crop pollen as revealed by a post hoc Tukey's test (significant *Ps *< 0.02). Data points are jittered horizontally and vertically

In floral surveys, numbers of flower taxa differed significantly among site types (*df* = 3, 37, *F *=* *14.0, *p *<* *.0001). Number of taxa at apple sites (9.2 ± 3.9; *N *=* *5) was not significantly different from blueberry (7.4 ± 3.0; *N *=* *11, *p *=* *.80) or cranberry sites (13.3 ± 3.8; *N *=* *12, *p *=* *.19), but had fewer taxa than fallow sites (16.8 ± 4.2; *N *=* *13, *p *=* *.002). Blueberry sites had fewer taxa than cranberry sites (*p *=* *.003) and fallow sites (*p *<* *.0001). Cranberry and fallow sites did not differ (*p *=* *.11; Figure 2).

**Figure 3:**
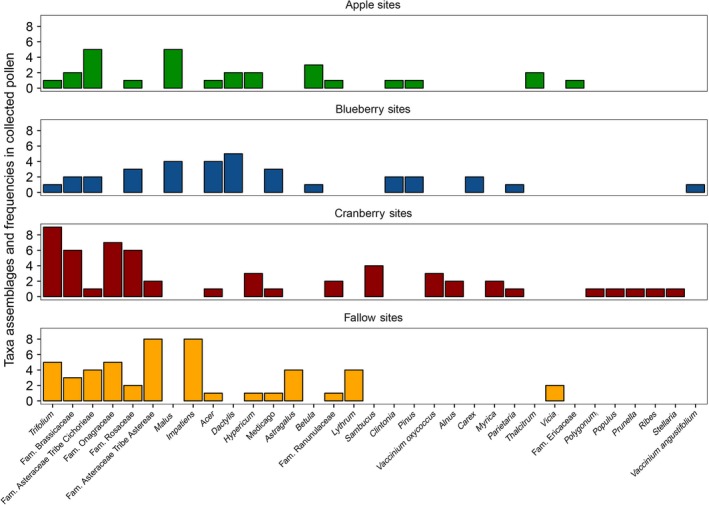
Frequency of floral source taxa identified in honey bee‐collected pollen from sites. Thirty‐three different pollen sources were identified, mostly to genus. Some taxa were common to all site types (e.g., *Trifolium* spp.), whereas some taxa were unique to one site type (e.g., *Vicia* spp. in fallow sites)

Thirty‐three plant taxa were identified in collected pollen from 27 sites, mostly to genus level (Figure 3). There were no significant differences among site types in diversity of pollen collected by honey bees (χ^2^(3) = 4.3, *p *=* *.20). Apple sites had 4.1 ± 0.8 taxa (*N *=* *9), blueberry 5.2 ± 1.9 (*N *=* *10), cranberry 5.1 ± 1.4 (*N *=* *14), and fallow 4.4 ± 1.2 (*N *=* *16). Diversity from floral surveys did not predict diversity in collected pollen (χ^2^(1) = 0.10, *p *=* *.70).

**Figure 4:**
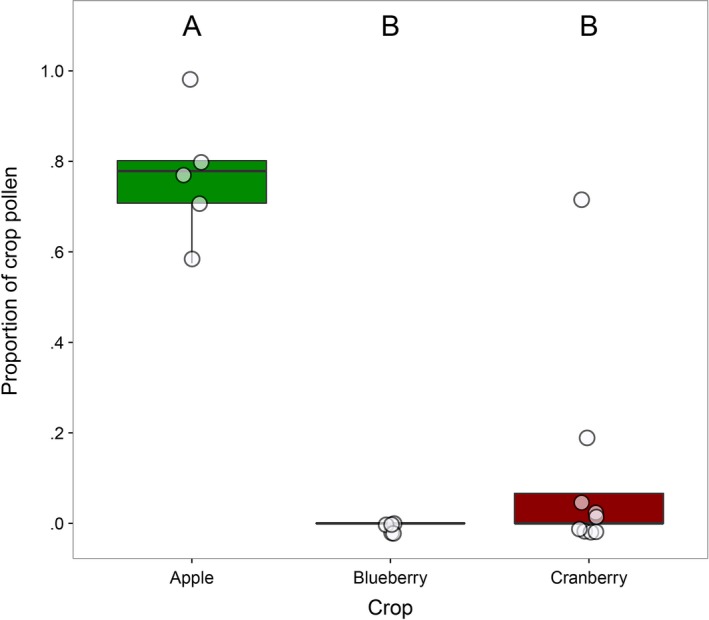
Proportion of honey bee‐collected pollen from crop flowers varied significantly among crop sites. Crops with different letters had statistically significant differences in proportion crop pollen as revealed by a post hoc Tukey's test (significant *Ps *< 0.0001). Data points are jittered horizontally and vertically

Proportions of pollen collected from crop flowers (e.g., apple pollen collected in apple sites) differed among crop sites (χ^2^(2) = 26.1, *p *<* *.0001; Figure 4). Apples sites had the highest proportion of pollen collected from crop flowers (0.74 ± 0.1; *N *=* *7), followed by cranberry (0.078 ± 0.20; *N *=* *14) and blueberry sites (0.0011 ± 0.0035; *N *=* *10) which did not differ.

### 3.2 Nutrition in pollen

**Figure 5:**
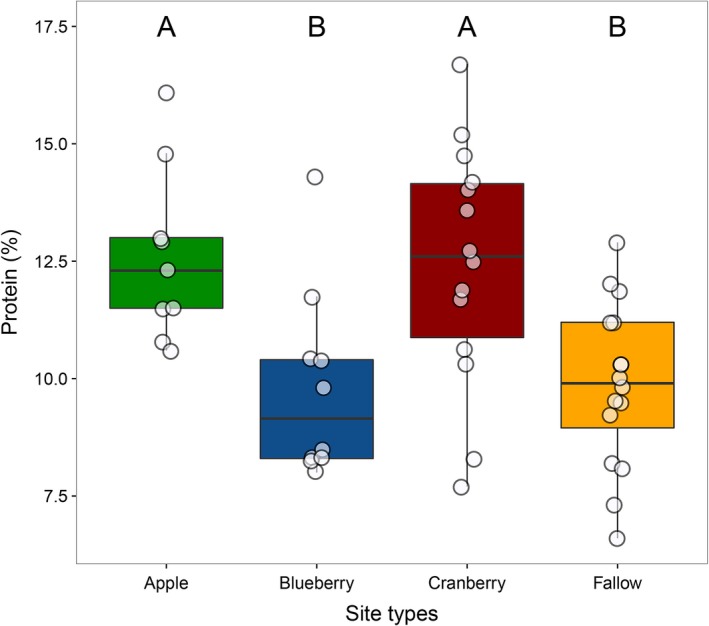
Percent protein of honey bee‐collected pollen differed significantly among site types. Sites with different letters had statistically significant differences in proportion crop pollen as revealed by a post hoc Tukey's test (significant *Ps *< 0.02). Data points are jittered horizontally and vertically

Percent protein of pollen differed significantly among sites (χ^2^(3) = 15.5, *p *=* *.002; Figure 5). Apple (12.61 ± 1.84; *N *=* *9) and cranberry (12.44 ± 2.58; *N *=* *14) sites had higher percent protein pollen than both blueberry (9.80 ± 2.02; *N *=* *10) and fallow sites (9.87 ± 1.74; *N *=* *16). Percent of all nine amino acids differed significantly among sites (χ^2^(3) range = 8.1 to 15.1, *p* range = .002 to .05; Fig. S1). Apple (*N *=* *9) and cranberry (*N *=* *14) sites trended higher in percent amino acids than both blueberry (*N *=* *10) and fallow sites (*N *=* *16).

### 3.3 Residues in pollen

**Figure 6:**
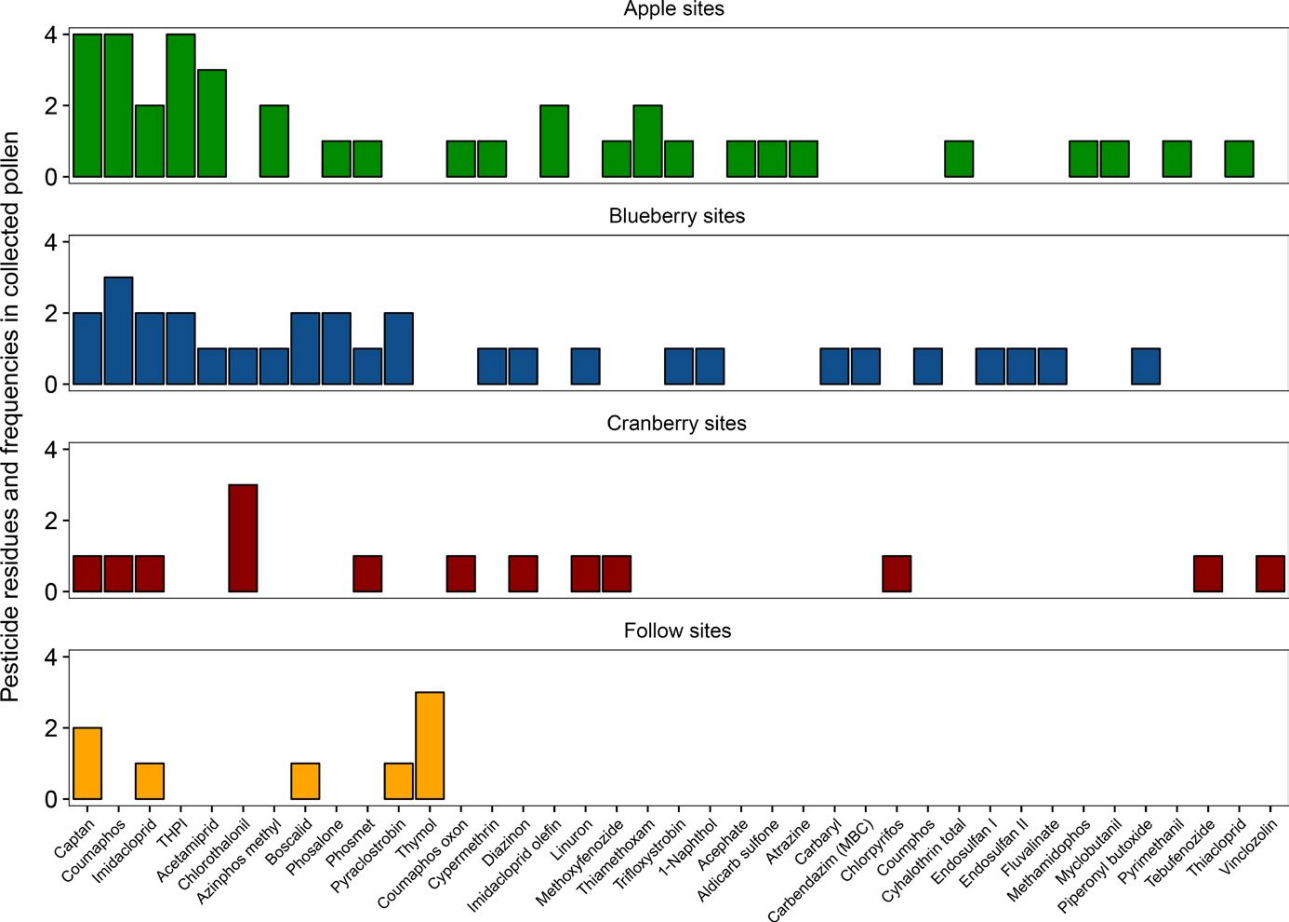
Frequency of pesticide residue detections in honey bee‐collected pollen from all colonies, stacked by site type. Thirty‐nine different residues were detected; some are breakdown products of others (e.g., coumaphos oxon from coumaphos). Some pesticides were detected in all site types (e.g., imidacloprid), whereas some pesticides were unique to one site type (e.g., chlorpyrifos in cranberry sites)

In 86 pollen samples, there were 269 separate detections of 39 different residues (Figure 6); 72 samples (83.7%) had at least one detection. Residues included 16 insecticides, 11 fungicides, five acaricides, two herbicides, one insecticide synergist, and four breakdown products (Table S1). All residues detected were below LD_50_, the amount needed to kill 50% of organisms exposed.

**Figure 7:**
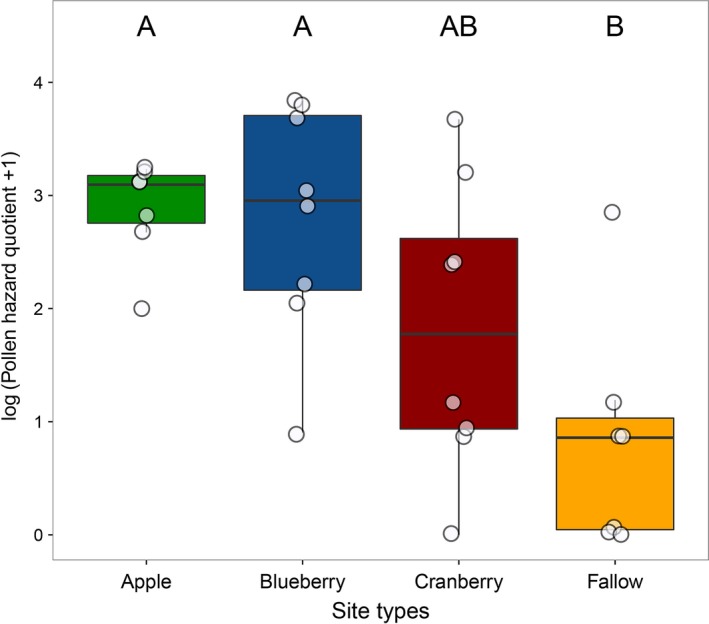
Pollen hazard quotients (PHQ) of pesticide residues in honey bee‐collected pollen differed significantly among site types. Site types with different letters had statistically significant differences in PHQ as revealed by a post hoc Tukey's test (*Ps *< 0.02). Data points are jittered horizontally and vertically

**Figure 8:**
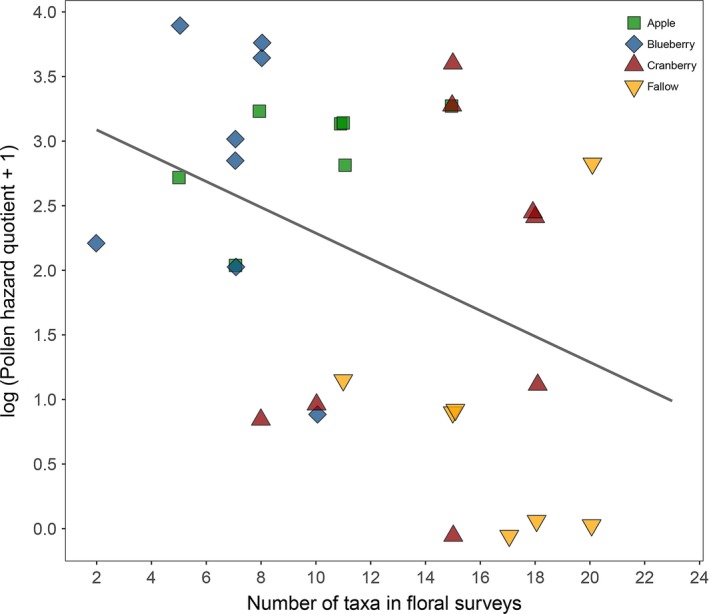
Pollen hazard quotient (PHQ) was significantly negatively correlated with number of taxa in floral survey around colonies. Data points are jittered horizontally and vertically

Number of pesticide residues detected in honey bee‐collected pollen differed significantly among site types (χ^2^(3) = 9.0, *p *=* *.03; pesticide amount was log‐transformed). Apple sites (5.6 ± 1.1; *N *=* *7) did not differ from blueberry (4.7 ± 4.1; *N *=* *8) or cranberry sites (2.7 ± 1.98; *N *=* *7), but had more residues than fallow sites (1.2 ± 0.5; *N *=* *6, *p *=* *.005). There were significant differences in PHQ among site types (χ^2^(3) = 13.6, *p *=* *.004; Figure 7). Apple (1,033.8 ± 621.7; *N *=* *7) and blueberry (2,520.8 ± 2,990.0; *N *=* *8) sites were not different from cranberry (854.3 ± 1,647.8; *N *=* *8) sites, but had higher PHQs than fallow sites (104.7 ± 264.8; *N *=* *7). Values of PHQ were negatively correlated with number of taxa in floral surveys (χ^2^(1) = 4.2, conditional *R* ^2^ = 0.66, *p *=* *.04; Figure 8).

## 4 DISCUSSION

Our results have a broad spatial and temporal scope that facilitates generalizations about honey bees pollinating apples, blueberries, cranberries, and fallow sites in the Maritimes and likely in other similar intensively managed agro‐environments. As expected, fallow sites had the greatest diversity of flowering plants because those sites were in uncultivated areas where species are not selectively excluded. However, we did not anticipate significant differences among the other site types in floral diversity; these differences suggest that either management practices in those crops differentially affect diversity or that the original abiotic and biotic conditions did so while simultaneously favoring sowing of specific crops. It is also possible that seasonal timing of these sites had an effect, with earlier sites (apple and blueberry) tending to have less diversity than later sites in the summer (cranberry and fallow).

We identified 33 different flower taxa in pollen collected by bees. Additional diversity likely occurred in a few pollen taxa that were not identified to species and in taxa seldom used by honey bees. This additional diversity is estimated in species accumulation curves, with none reaching a clear asymptote (Fig. S2). Moreover, because of intraspecific diversity in plant quality, species‐level diversity is an incomplete metric of diet diversity and quality. In any case, our data show that honey bees used four to five different taxa as pollen sources at any particular time throughout the pollination season (also see Singh & Singh, 1996); this was less than half the floral diversity we captured in concomitant floral surveys. In addition, although there were significant differences in flower diversity among site type surveys, they did not affect the diversity of pollen that honey bees collected. This suggests that within the crops we sampled, honey bees found enough floral diversity to forage on, because they used fewer taxa than were available around them at our sites. However, we also observed that some crops may not provide sufficient protein; this could be remedied by planting pollinator‐friendly plants adjacent to or within crops (Decourtye, Mader, & Desneux, 2010). This could be even more important for honey bees pollinating larger monoculture crops where travel to alternate pollen sources may be too energetically costly.

Pollen collections and floral surveys were performed in one or two days at each site, giving a “snapshot” of what was available to, and used by, honey bees. Additional diversity in honey bee diets would almost certainly be observed over longer intervals; however, honey bees are deployed in various crops for relatively brief intervals, so our sampling likely captures the majority of variation that occurs in this landscape and within the management regime of beekeepers in much of North America. Additionally, honey bees do not forage in rain or cold temperatures, and this further limits the number and quality of plants that honey bees are likely to visit. Nonetheless, summer 2011 was fairly wet and cool (Environment Canada 2012), meaning that pollen was possibly less diverse than in other years.

In apple sites, the majority of pollen in every colony came from apple flowers, suggesting that it may be favored by honey bees because it is nutritious (also see Laverty & Hiemstra, 1998), or as is detailed below, easy for them to collect. In contrast, only one honey bee colony collected blueberry pollen, and then, it made up only ~ 1% of all pollen (also see Dogterom & Winston, 1999; Javorek, MacKenzie, & Vander Kloet, 2002; MacKenzie, 1994). Similarly, proportions of pollen from cranberry were quite low compared to proportions from apple flowers. The low proportions of blueberry and cranberry pollen could be ascribed to the foraging style of honey bees. Blueberry and cranberry both have bell‐shaped flowers that are best served by buzz pollinators, such as bumble bees, wherein thoracic muscles are vibrated to release pollen grains (Javorek et al., 2002). Honey bees are not buzz pollinators, and this could be an obstacle to pollen collection. An additional explanation for the low amount of *Vaccinium* spp. pollen is that openings in pollen traps may not be narrow enough to remove those pollen loads. *Vaccinium* spp. pollen grains, especially of blueberry pollen, are not easily packed into large loads by honey bees and may not be dislodged by traps (Girard, Chagnon, & Fournier, 2012; Hodges, 1974). This does not mean that honey bees do not pollinate blueberries and cranberries, only that pollen is not commonly collected by honey bees, or was missed because of trap bias. In fact, although *Vaccinium* pollen may not make it to the corbiculae of honey bee foragers, their bodies can be covered by a significant amount of pollen grains that pollinate flowers as nectar is collected (Dogterom & Winston, 1999). In contrast, apple flowers have relatively open flowers whose anthers dehisce naturally (Williams & Brain, 1985), reducing collection effort for honey bees.

Both apple and cranberry site pollen had higher protein levels than blueberry and fallow pollen. A similar trend was seen for all of the nine amino acids analyzed. There may be a trade‐off between the effort of collecting cranberry pollen and its nutrient value compared to more easily collected pollen of other flowers in bloom. In blueberry sites, where almost no pollen in traps was from blueberry flowers, other forage may be of higher quality, easier to collect, or a combination of the two. However, the low percentage of protein compared with the other two crop sites also suggests that pollen forage available to honey bees around blueberries is of lower nutritional quality. We were surprised at the relatively low amount of protein in fallow pollen; possibly all forage available at this time is of low nutritional quality, at least compared with pollen in apple and cranberry sites. Alternatively, crop flowers may have more nutritious pollen because of fertilizer application (Atasay, Akgül, Uçgun, & Şan, 2013). It is possible that pollen from blueberry and cranberry flowers has similar protein levels, because they are in the same genus (Somerville & Nicol, 2006). Our results suggest that honey bees have lower protein available to them at blueberry (see also Girard et al., 2012) and fallow sites, but experimental data are required before recommending management for supplementation during these times.

Mullin et al. (2010) documented residues in 23 states and one province and reported nearly triple the total residues detected in this study (98 compared to our 39). Eighty‐nine percent of samples in our study had at least one residue detected, similar to 87.7% of samples in the French study (Chauzat et al., 2006), whereas 100% of pollen samples in the American study had at least one residue detected (Mullin et al., 2010). We also observed only 3.9 residues within crop sites compared to 7.1 reported by Mullin et al. Some of these differences are likely due to differences in geographic scales of our study, lower transport distances of beekeeping operations, and a lower diversity of crops that our operations experience, and hence pesticide exposures. There is a difference between the United States and Canada in total amount of pesticides used. In 2007, total insecticide active ingredient use in the United States was 78.5 million kg and in 2008 Canadian usage was 3.0 million kg (ratio of 26.5; FAO 2016). Compared to area of cultivated lands (United States 157.8 million ha, Canada 35.2 million ha), the United States used an average of 0.5 kg/ha and Canada used an average of 0.09 kg/ha (ratio of 4.5; Statistics Canada 2011; USDA 2012).

Pollen in apple sites had higher numbers of residues than fallow sites, but neither differed from blueberry or cranberry sites. Additionally, apple and blueberry sites had the highest PHQ values, though not significantly higher than cranberry. The high number of residues and associated PHQ in apple pollen were not surprising because, presumably, crop flowers would have higher application rates of pesticides, and a high proportion of pollen in apple sites was from apple flowers. However, there was almost no blueberry pollen trapped in blueberry sites. One explanation for high residue and PHQ levels but low crop flower proportion is pesticide drift to noncrop flowers around blueberry sites during application or runoff. As expected, fallow sites had few residues because these sites are not sprayed directly.

We found a significant negative correlation between floral diversity and PHQ. It may be that reduced pesticide application is associated with higher floral diversity. This suggests that higher floral diversity at pollination sites reduces pesticide exposure for honey bees. However, we found no significant correlation between diversity of pollen sources and numbers of residues detected. Thus, floral diversity does not affect diversity of residues directly in pollen, but could have an indirect effect. This indirect effect could be due to fewer pesticides being encountered by nectar foragers on nearby flowers; when pollen is collected by pollen foragers, honey from their honey stomachs is added to pollen pellets (Winston, 1991). If fewer residues are in honey, it would mean fewer residues would be transferred to pollen, lowering the total number of residues present in pollen. This warrants more testing. Testing honey bee foragers themselves, and for presence of residues in honey stomachs, could address this unresolved issue. A related question from this study is how traditionally in‐hive chemicals (i.e., coumaphos, fluvalinate) got in pollen that had been trapped outside of colonies, as has also been reported elsewhere (Chauzat et al., 2006; Mullin et al., 2010).

None of the detected residues were at, or above, LD_50_ concentrations. This suggests that honey bees in this study were not in danger of acute poisoning by pesticide exposure, but this does not exclude the possibility of sublethal effects, including poor queen performance (e.g., Frost et al., 2013; Williams et al., 2015). Possible sublethal effects of residues detected in this study should be explored to evaluate hazards that pesticide exposure present to commercial honey bees. Moreover, there are thousands of potential synergies among residues that have scarcely been evaluated. For example, synergistic effects have been identified in combinations of chlorothalonil and coumaphos, and chlorothalonil and fluvalinate (Zhu, Schmehl, Mullin, & Frazier, 2014). One or both of these combinations were found in each of our crop site types.

Two of the most common five pesticides detected, imidacloprid, and acetamiprid, are neonicotinoids. Two additional neonicotinoids were detected less frequently: thiamethoxam and thiacloprid. Neonicotinoids have been under particular scrutiny in recent years for their implication in honey bee losses, and their use as seed treatments has been partially restricted in the European Union (European Commission, 2013). That neonicotinoids were detected in pollen with such regularity is concerning and bears monitoring.

Our experimental design does not allow us to distinguish explicitly between the separate effects of time of year and site type. However, our design is broadly representative of what honey bees would be exposed to in commercial use. Moreover, asynchrony of flowering seasons of the various crops precludes a design that could separate time of year from site type.

Our results show adequate levels of protein and essential amino acids and no lethal levels of residues in pollen. We believe that an important next step for a greater understanding of pesticide exposure in honey bees is to extend the scope of future studies to include samples of other honey bee‐collected substances, such as nectar, honey, propolis, and water. Increasing sample sizes of pollen collected from crop flowers could allow more detailed analyses of pesticide exposures during pollination seasons. Finally, possible sublethal effects of residues detected in this study should be explored to evaluate hazards that pesticide exposure present to commercial honey bees.

## CONFLICT OF INTEREST

None declared.
