# Supplementary material for: Honey bee‐collected pollen in agro‐ecosystems reveals diet diversity, diet quality, and pesticide exposure
Source: Ecol Evol. 2017 Aug 5;7(18):7243–53. doi: 10.1002/ece3.3178 (PMC5606875; doi:10.1002/ece3.3178)
Supplement: Supplementary file 1 [file ECE3-7-7243-s001.docx]

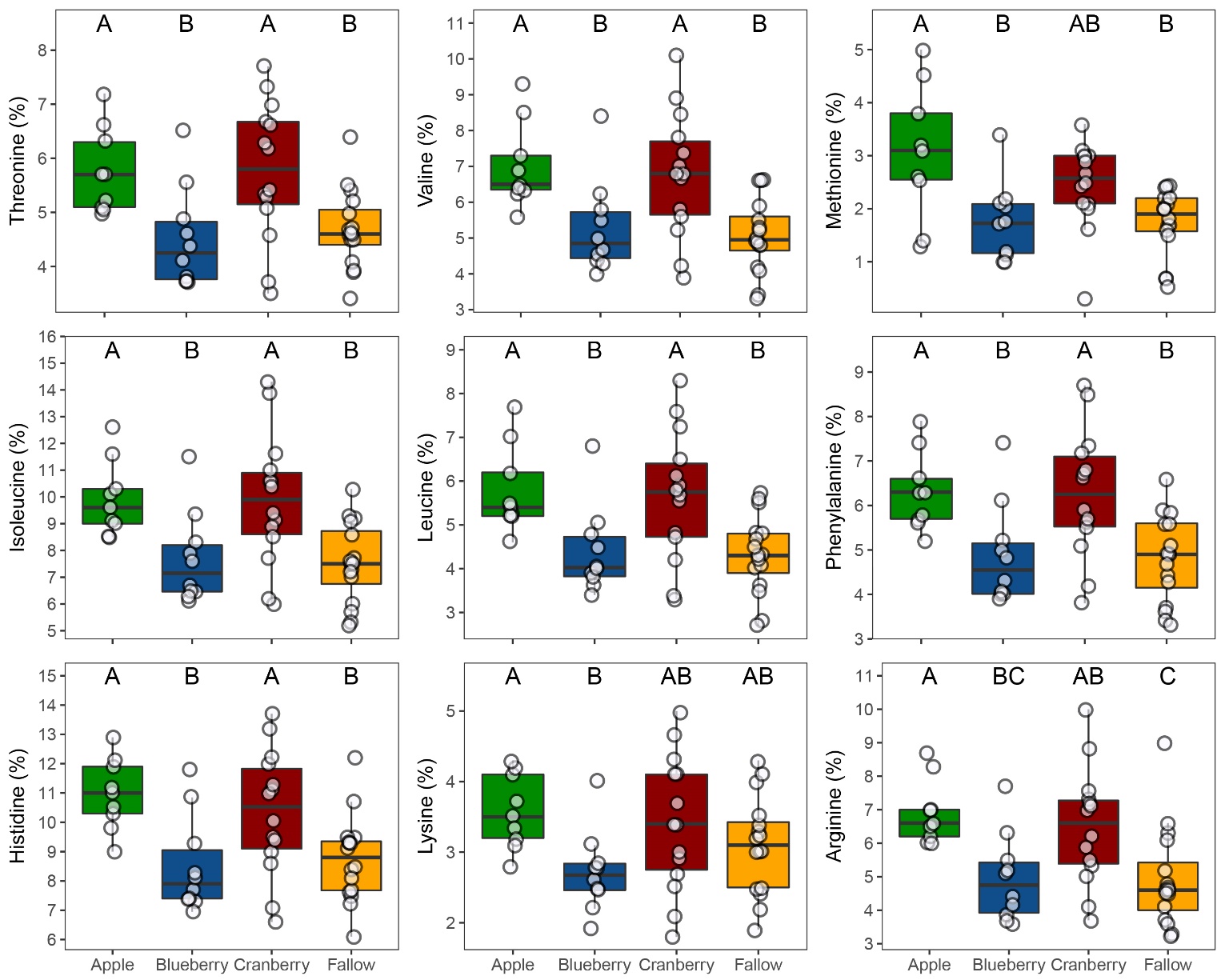


Fig. S1. Percent of nine essential amino acids of honey bee-collected pollen differed significantly among site types. Sites with different letters had statistically significant differences in percent amino acids as revealed by post hoc Tukey’s test (*Ps* range < 0.04 to < 0.03). Data points are jittered horizontally and vertically.
