# Supplementary material for: Honey bee‐collected pollen in agro‐ecosystems reveals diet diversity, diet quality, and pesticide exposure
Source: Ecol Evol. 2017 Aug 5;7(18):7243–53. doi: 10.1002/ece3.3178 (PMC5606875; doi:10.1002/ece3.3178)
Supplement: Supplementary file 2 [file ECE3-7-7243-s002.docx]

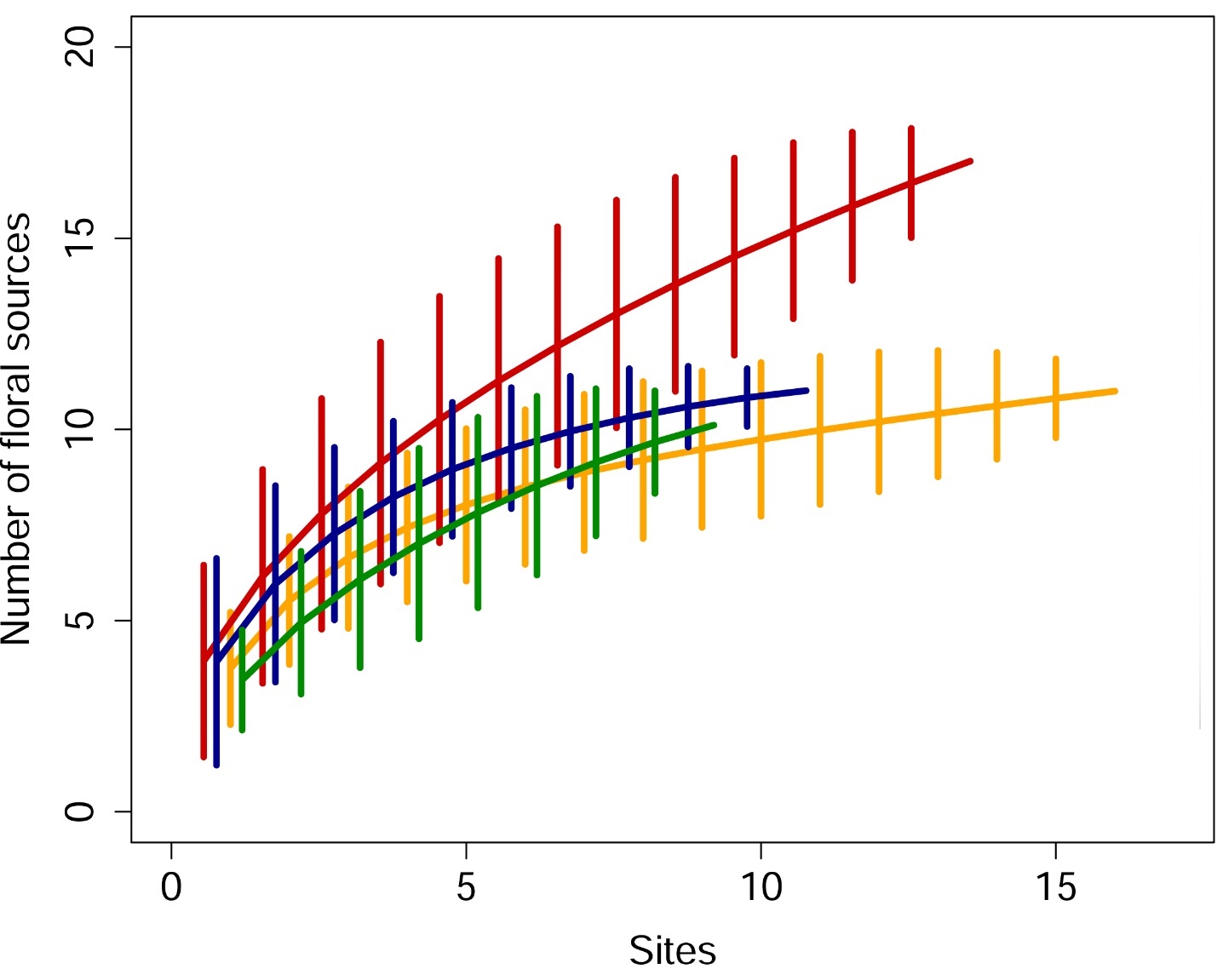


Fig. S2. Taxon accumulation curves for honey bee-collected pollen by site type. Vertical lines are 95% confidence intervals. Curves are jittered horizontally.
