# Supplementary material for: Honey bee‐collected pollen in agro‐ecosystems reveals diet diversity, diet quality, and pesticide exposure
Source: Ecol Evol. 2017 Aug 5;7(18):7243–53. doi: 10.1002/ece3.3178 (PMC5606875; doi:10.1002/ece3.3178)
Supplement: Supplementary file 3 [file ECE3-7-7243-s003.docx]

Table S1. Summary of pesticide residue results from all sites (n=86). Detections and LC50s reported as parts per billion (ppb). Residue types: B = Breakdown product, I = Insecticide, H = Herbicide, F = Fungicide, A = Acaricide, IS = Insecticide Synergist (n.d. = no data on toxicity; * = breakdown product of carbaryl, † = breakdown product of coumaphos, ‡ = breakdown product of imidacloprid, ¶ = breakdown product of captan; EPA 2012; Kegley *et al.* 2012; NCAP 2012; NPIC 2012).

|  |  |  |  | | | | Reported oral LC_50_ (ppb) | |
| --- | --- | --- | --- | --- | --- | --- | --- | --- |
| Residue | **Type** | **Detections** | **Mean** | **S.D.** | **Min.** | **Max.** | **Low** | **High** |
| 1-Naphthol | B* | 4 | 167.2 | 43.9 | 103.0 | 202.0 | n.d. | n.d. |
| Acephate | I | 1 | 70.7 | N/A | 70.7 | 70.7 | 38461 | n.d. |
| Acetamiprid | I | 16 | 9.9 | 9.2 | 2.4 | 27.4 | 558846 | n.d. |
| Aldicarb sulfone | I | 2 | 3.7 | 2.3 | 2.1 | 5.3 | 10961 | n.d. |
| Atrazine | H | 2 | 7.9 | 0.3 | 7.7 | 8.1 | 3730769 | n.d. |
| Azinphos methyl | I | 7 | 176.5 | 111.5 | 34.8 | 381.0 | 129230 | n.d. |
| Boscalid | F | 7 | 447.2 | 301.8 | 16.3 | 820.0 | 6384615 | n.d. |
| Captan | F | 35 | 2842.7 | 3117.2 | 62.0 | 12000.0 | 384615 | n.d. |
| Carbaryl | I | 4 | 1286.8 | 285.1 | 927.0 | 1570.0 | 8846 | 48846 |
| Carbendazim (MBC) | F | 4 | 119.5 | 45.4 | 71.9 | 180.0 | 1923076 | n.d. |
| Chlorothalonil | F | 14 | 7520.4 | 14017.1 | 465.0 | 50500.0 | 6972692 | n.d. |
| Chlorpyrifos | I | 11 | 11.9 | 16.6 | 3.6 | 61.2 | 2269 | n.d. |
| Coumaphos | A | 18 | 33.7 | 51.9 | 2.1 | 211.0 | 76923 | 576923 |
| Coumaphos oxon | B† | 2 | 13.4 | 3.4 | 11.0 | 15.8 | n.d. | n.d. |
| Cyhalothrin total | I | 1 | 29.9 | N/A | 29.9 | 29.9 | 21923 | n.d. |
| Cypermethrin | I | 3 | 20.9 | 14.3 | 4.8 | 32.2 | 6615 | n.d. |
| Diazinon | I | 9 | 11.2 | 12.6 | 2.2 | 35.9 | 9615 | n.d. |
| Dicofol | A | 1 | 2.8 | N/A | 2.8 | 2.8 | 469230 | n.d. |
| Endosulfan I | I | 1 | 65.2 | N/A | 65.2 | 65.2 | 188461 | n.d. |
| Endosulfan II | I | 1 | 144.0 | N/A | 144.0 | 144.0 | n.d. | n.d. |
| Fluvalinate | A | 9 | 34.1 | 13.8 | 17.9 | 58.7 | 7692 | 46153 |
| Imidacloprid | I | 25 | 6.0 | 7.2 | 1.0 | 30.4 | 150 | 19230 |
| Imidacloprid olefin | B‡ | 5 | 108.2 | 54.8 | 52.4 | 197.0 | n.d. | n.d. |
| Linuron | H | 2 | 140.2 | 162.3 | 25.5 | 255.0 | 4648461 | n.d. |
| Methamidophos | A | 2 | 14.4 | 5.7 | 10.4 | 18.4 | 52692 | n.d. |
| Methoxyfenozide | I | 13 | 438.4 | 765.3 | 17.9 | 2520.0 | 3846153 | n.d. |
| Myclobutanil | F | 1 | 85.7 | N/A | 85.7 | 85.7 | 13923076 | n.d. |
| Phosalone | A | 5 | 345.2 | 304.7 | 82.4 | 710.0 | 169230 | n.d. |
| Phosmet | I | 5 | 822.6 | 866.1 | 15.1 | 2030.0 | 19230 | 38461 |
| Piperonyl butoxide | IS | 3 | 48.7 | 21.6 | 23.8 | 62.0 | 423076 | n.d. |
| Pyraclostrobin | F | 6 | 85.6 | 56.5 | 11.8 | 141.0 | 3846153 | n.d. |
| Pyrimethanil | F | 2 | 3.6 | 0.6 | 3.1 | 4.0 | 3846153 | n.d. |
| Tebufenozide | I | 3 | 1876.7 | 491.0 | 1540.0 | 2440.0 | 9000000 | n.d. |
| Thiacloprid | I | 1 | 1.4 | N/A | 1.4 | 1.4 | 666153 | 1455000 |
| Thiamethoxam | F | 3 | 6.5 | 2.0 | 5.2 | 8.8 | 192 | 3384 |
| THPI | B¶ | 25 | 3250.0 | 3418.0 | 484.0 | 17500.0 | n.d. | n.d. |
| Thymol | F | 9 | 72.3 | 13.9 | 46.6 | 85.5 | n.d. | n.d. |
| Trifloxystrobin | F | 4 | 114.6 | 103.4 | 17.1 | 251.0 | 7692307 | n.d. |
| Vinclozolin | F | 3 | 2.9 | 1.0 | 2.1 | 4.0 | 3846153 | n.d. |
